# Cellulose transparent conductive film and its feasible use in perovskite solar cells

**DOI:** 10.1039/c9ra01301f

**Published:** 2019-03-22

**Authors:** Xiaojuan Ma, Qidu Deng, Lu Wang, Xin Zheng, Shunshun Wang, Qinhua Wang, Lihui Chen, Liulian Huang, Xinhua Ouyang, Shilin Cao

**Affiliations:** College of Material Engineering, Fujian Agriculture and Forestry University Fuzhou 350002 China tomyouyangxh@outlook.com scutcsl@163.com

## Abstract

A transparent conductive Ag nanowire (AgNW)-regenerated cellulose film (RCF) was prepared and has been proposed to be used as an anode for perovskite solar cells. The HNO_3_ treatment was used for solving the dilemma between optical transparency and conductivity caused by the AgNW introduction; in addition, the bonding strength between AgNWs and cellulose film was enhanced substantially *via* HNO_3_ treatment. Accordingly, the AgNW-RCF with a AgNW size of 15 μm × 85 nm and density of 0.36 g m^−2^ shows impressive conductivity and transparency, with a sheet resistance of 29 Ω □^−1^ and a transmittance of 80% at a wavelength of 550 nm. Perovskite solar cells incorporating such AgNW-RCF anodes exhibited a cell performance with a *V*_oc_ of 1.02 V, *J*_sc_ of 9.58 mA cm^−2^, FF of 45.8% and PCE of 4.49%.

## Abstract 2

A transparent conductive Ag nanowire (AgNW)-regenerated cellulose film (RCF) was prepared and has been proposed to be used as an anode for perovskite solar cells.

## Graphical abstract

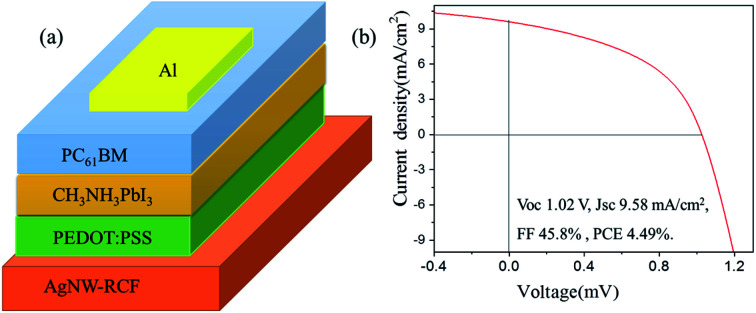

## 1 Introduction

High efficiency and eco-friendly photovoltaics have been persistently pursued over the past decade to realize renewable solar energy conversion,^1,2^ where, substrates play an important role as the foundation or functional component for the solar cell. Among the substrates, cellulose based materials are emerging as a useful substrate in electronic devices due to cellulose's earth abundance, remarkable optical and other properties such as thermal stability, nontoxicity and biodegradability.^3–8^

It is well known that optical transparency and electrical conductivity of the electrodes are key parameters that affect the power conversion efficiency (PCE) significantly.^9–11^ As for a thin photovoltaic film, optical management is important for harvesting light while ensuring high efficiency. High transparency is a relatively simple task for preparation cellulose based film. Films or nanopapers prepared from regenerated cellulose,^12,13^ rational mixture of NCC and fiber,^4^ NFC,^14^ cellulose–CMC composite^15^ and TEMPO-oxidized nanocrystalline cellulose^16^ are always transparent. Whereas, the two mutually opposing properties of conductivity and transparency combined in one material is a challenging task for fabricating electronic and optoelectronic devices. However, several attempts referring to the conductive transparent cellulose film/paper including sputtering ITO onto cellulose regenerated film,^17^ precipitation of silver nanowires (AgNW) or polydopamine^16,18,19^ on cellulose nanopaper and assemble of Au/polythiophene/cellulose sheet^20^ have been developed. In addition, these conductive cellulose films and some cellulose derivative have been studied as a substrate in fabrication of dye-sensitized solar cells.^17,21,22^ There is little done in the area of transparent conductive regenerated cellulose film; to the best of our knowledge, the thin film prepared from bacterial cellulose/AgNWs composite exhibited an acceptable transparency ∼80% and a good conductivity 7.46 Ω □^−1^;^23^ while the transparent films from remaining wood microstructures and AgNW showed a slight lower transparency ∼75% and a good conductivity 11 Ω □^−1^.^24^

In this work, regenerated cellulose film was prepared from cellulose dissolution in ionic liquid and regeneration from a water bath. A modified polyol synthesis method was adopted to grow AgNW on the AgCl core with a controlled size. The AgNWs were precipitated onto the cellulose film by the means of slow centrifugal settling. The transparency loss caused by AgNW introduction was mitigated by post HNO_3_ treatment; which could consume some of the junctions of AgNW network. After that, the thin film with a transparency of 80% and sheet resistance of 29 Ω □^−1^ was used as an anode for perovskite solar cells fabrication. The photovoltaic performance of this solar cell was discussed.

## 2 Experimental

### 2.1 Materials

Commercial coniferous dissolving pulp with α-cellulose content of 95% and DP of 500 was used for preparation of regenerated cellulose film. 1-Allyl-3-methyl imidazolium chloride and lead(ii) iodide (PbI_2_) was purchased from Aladdin Co. Ltd, Shanghai, China. Silver nitrate (AgNO_3_), isopropyl alcohol (C_3_H_8_O) and *N*,*N*-dimethylformamide (DMF) was purchased from Sinopharm Chemical Reagent, Co., Ltd., (Shanghai, China), polyvinyl pyrrolidone (PVP) and polyethyleneimine (PEI) was purchased from Aladdin Bio-Chem Technology Co., Ltd. Zinc chloride (ZnCl_2_, AR) and ethylene glycol (EG) was purchased from Shanghai Macklin Biochemical Co., Ltd. The rest chemicals used for perovskite solar cells fabrication were provided by Dalian qiseguang solar energy technology Co., Ltd., (Dalian, China).

### 2.2 Preparation of cellulose films

Commercial coniferous dissolving pulp with α-cellulose content of 95% and DP of 500 was used for preparation of regenerated cellulose film. Cellulose (3%) was dissolved in 1-allyl-3-methyl imidazolium chloride and regenerated from water bath. The detailed procedure was described in our previous work.^25^ The final cellulose film was with a roughness of *R*_a_ 2.36 nm and a high light transmittance of ∼90%.

### 2.3 Preparation of silver nanowire (AgNW)

The silver nanowire (AgNW) was prepared using EG reduction method. EG 40 mL and PVP with high molecular weight (DP 58 000) 1.2 g was mixed thoroughly with magnetic stirring and then 0.05 g AgCl was added into the mixture when the temperature was reached to 170 °C (oil bath). 10 min later, 0.4 g AgNO_3_ was added to start the growth of AgNW. After 30 min reaction, PEI was added and the reaction was kept for 10 min. After that, the AgNW was centrifugal precipitated from the mixture with 5000 rpm for 20 min followed by isopropyl alcohol washing for 3 times. The final AgNW with a high purity was with a length of 8–20 μm (average 15 μm) and a diameter of 50–95 nm (average 85 μm).

### 2.4 Conductive cellulose film preparation

100 mL isopropyl alcohol was added into the centrifugal beaker padded with a cellulose film at the bottom. AgNW was added slowly into the beaker followed by an ultrasonic oscillation for uniform dispersion. The AgNW was precipitated onto the cellulose film by the means of low speed centrifugation. After the conductive film was taken out of the beaker, the film was fixed on to a PVA plate and vacuum dried for 1 h at 80 °C. Subsequently, the dried conductive film was treated by HNO_3_ and washed with water several times. The final film was vacuum dried for the analysis and application.

### 2.5 Fabrication of perovskite solar cells

The precursor solution was prepared from solution mixture of PbI_2_ (1 mol L^−1^) and DMF. Perovskite solar cell was fabricated on AgNW-RCF substrate. The AgNW-RCF was cleaned in sequence by N_2_ purge and ultraviolet ozone treatment. PEDOT:PSS solution was spin-coated onto the AgNW-RCF which was fixed onto a PVA plate (Spin coater, Laurell WS-400B-6NPP-LITE, USA, the rotate speed was controlled at 3000 rph; the thickness of the PEDOT:PSS film was controlled at 30–40 nm). The combined film was treated at 100 °C for 10 min. After that, PbI_2_ precursor was spin coated onto the PEDOT:PSS film followed by annealing treatment for 20 min at 80 °C. 45 mg mL^−1^ CH_3_NH_3_I was coated (the thickness was controlled at ∼400 nm) and followed by annealing treatment at 70 °C. PC_61_BM was dissolved in chlorobenzene solution to prepare a solution with a concentration of 4%. After 30 min, the PC_61_BM was spin coated on the perovskite in the same method. Finally, gold (Au) electrode (100 nm) was thermally evaporated under high vacuum condition. The effective area of the prepared planar perovskite solar cell is 0.06 cm^2^.

### 2.6 Characterizations

The surface roughness of the regenerated cellulose film was determined by tapping-mode atomic force microscope (AFM, 5500LS, Agilent, USA) measurements. A scanning electron microscope (SEM) (FEI Nova NanoSEM 230, Holland) was used to observe the surface of the regenerated cellulose films and the AgNW at an accelerating voltage of 15 kV. Prior to the field-emission SEM test, the surface of the films was coated with gold by a sputter coater (E-1010, Hitachi, Japan). The transmittance of the films was determined using a UV-visible spectrophotometer (Agilent 8453, USA). The sheet resistance of the conductive film was determined by a Four-probe resistivity tester (Keithley2400, USA). Each sample was measured 10 points randomly, and the average value of resistance was obtained.

The bonding strength of the AgNW and RCF was inferred by the conductivity after 3 M tape test; the method was used for evaluating the resistance of a coating to detachment from a substrate (GB 9286). The current density–voltage (*J*–*V*) curves were measured using a Keithley 2612A source measurement unit, and photovoltaic performance was measured under an illumination intensity of 100 mW cm^−2^ using a San-Ei solar simulator at AM 1.5G conditions at 25 °C.

## 3 Results and discussion

### 3.1 AgNW introduction for imparting RCF with conductivity

**Fig. 1:**
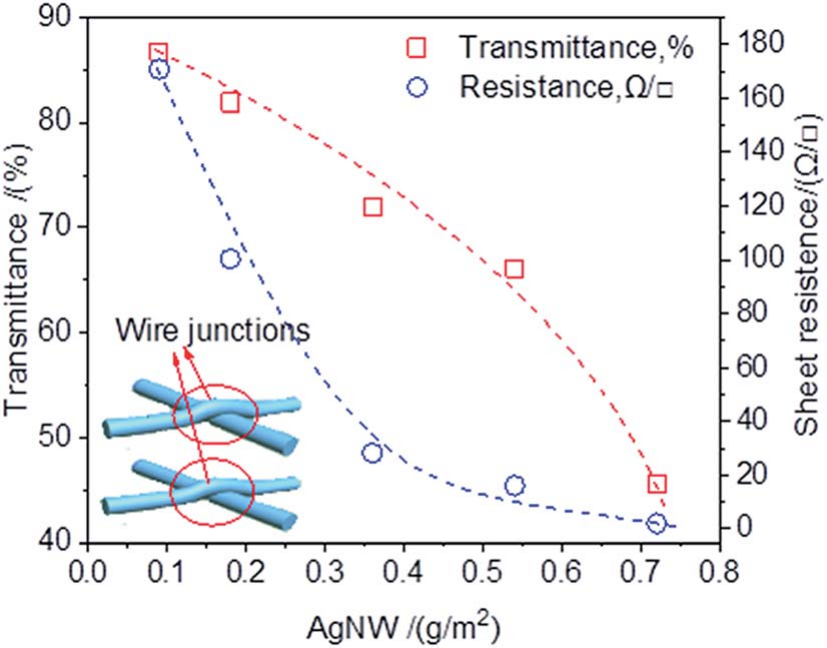
The effects of AgNW density on the transmittance and sheet resistance.

AgNW deposited onto the RCF substrate with the expectation that the AgNW networks act as a high conductivity electron pathway. Fig. 1 shows that AgNW density had a significant effect on the sheet resistance and optical transmittance of the AgNW-RCF. An increase of AgNW density are favorable for conductivity enhancement but at an expense of transparency loss; however, rising the AgNW to 0.36 g m^−2^ or higher, the conductivity was improved slightly but with a sharp decrease of transmittance.

As AgNW interweave formed during the deposition onto the RCF films, the network junctions were generated (the inset picture shown in Fig. 1), resulting more roughness surface of the AgNW-RCF; especially for the AgNW with large diameter. It is generally accepted that roughness is one of the parameters for determining the transparency of the film. For example, a nanopaper and plastic with low surface roughness (5 nm) have an extremely high optical transparency (90% at 550 nm), while rough paper (surface roughness of 5000–10 000 nm) has a lower optical transparency of 20%.^8^ The scattering effect on light initiated by the AgNW is not conducive to the improvement of light transmission performance of the films. In addition, with the increase of AgNW, the numbers of the wires junctions increased, which will lead a lingering conductivity enhancement. It is reported that resistances of Ag nanowires junction also significantly decreases the film conductivity.^26,27^

By contrast, the transmittance of the conductive film decreased more rapidly with increasing of AgNW density than the film from slice wood/AgNW and bacterial cellulose/AgNW.^23,24^ The diameter of the AgNW used in this study was larger, and the length was shorter than those were used in the reported literature.^23,24^ The AgNW with bulky structure could form a looser conductive wire network with weak connect between the AgNW, which are detrimental for the conductivity. Moreover, the thick AgNW coatings on the cellulose film negatively affected the film transmittance.

### 3.2 HNO_3_ treatment for improving the optical transmittance

**Fig. 2:**
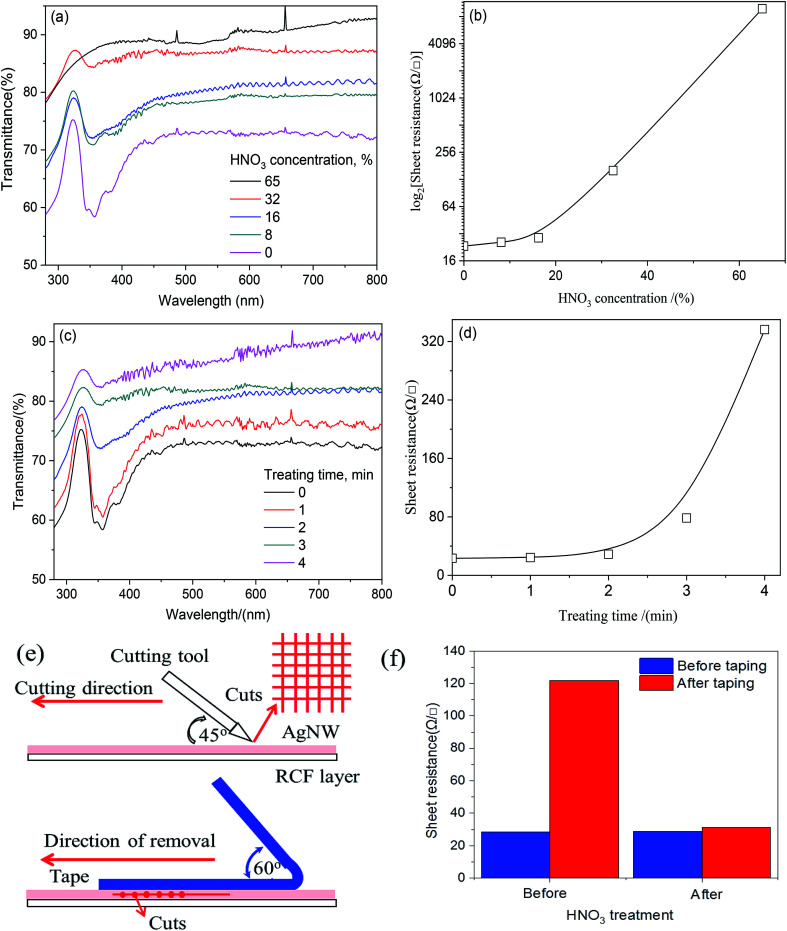
Gradual increase of HNO_3_ concentration (a) and treatment time (c) can improve the optimal transmittance (a), but are much damaged for the conductivity (b) and (d). Tape test mechanism (e) and sheet resistance of the AgNW-RCF before and after treatment (f). The starting sample was with AgNW content of 0.36 g m^−2^, sheet resistance 28 Ω □^−1^; transmittance 80%.

As discussed above, AgNW introduction could effectively enhance the conductivity, but the transmittance reduction caused by AgNW or junctions was remarkable. In order to enhance the transparency the AgNW-RCF, HNO_3_ treatment was used for decreasing the AgNW wire junction effects. Fig. 2a shows the HNO_3_ concentration effects on the transmittance, it is apparent that concentrated HNO_3_ treatment is favorable for transmittance improvement. However, it is detrimental for conductivity especially when the concentration is higher than 16% (Fig. 2b). It is possible that the HNO_3_ (0–16%) reacts with debris and junctions from AgNW network; resulting an improvement of transparency and conductivity. Other explanation is that HNO_3_ treatment would cause AgNWs merging with the other nanowires in the junction as well as hot pressing. Additionally, the AgNW consumption after reaction would change the AgNW structure with a slim one, which is favorable for the transparency and conductivity.

**Fig. 3:**
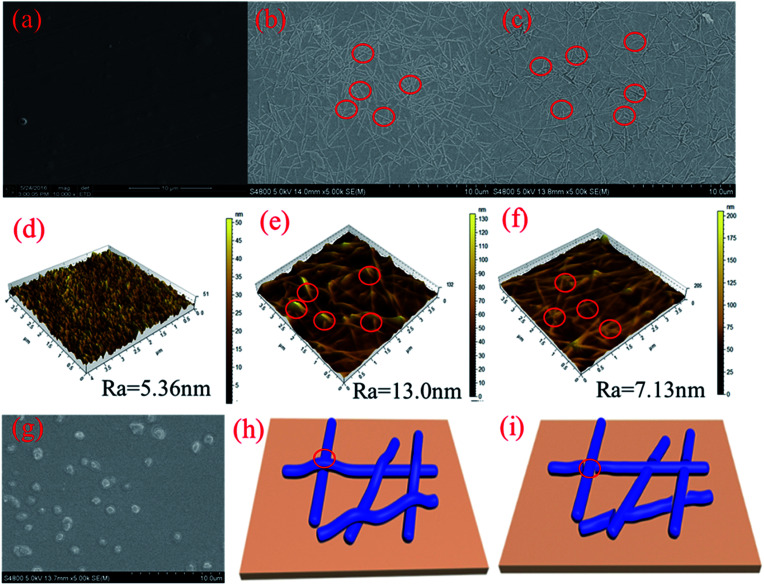
SEM and AFM image of the RCF (a and d), AgNW-RCF before (b and e) and after (c and f) HNO_3_ treatment (16%, 2 min), concentrated HNO_3_ (32%) treatment (g). Model of the wire junction before (h) and after (i) treatment.

In this range, the sheet resistance was slightly increased from 28 to 29 Ω □^−1^, while the transmittance increased greatly from 71 to 80%. HNO_3_ treatment with higher concentration (higher than 16%) would seriously damage to the network structure and therefore extremely high sheet resistance resulted (Fig. 3g). To be evident, the absorbance at 320 nm assigned to the Ag almost disappeared when the concentration increased to 65%, as shown in Fig. 2a. Treatment time also had effects on the conductivity and transmittance. Long time reaction would promote Ag consumption and be inferior to the conductivity. The results from Fig. 2c and d showed that control the reaction time within 0–2 min can obviously enhance the transmittance without conductivity loss.

The bonding strength was referred by the sheet resistance after 10 times 3 M tape test. Fig. 2e and f shows the cutting method and the sheet resistance of the AgNW-RCF after 3 M tape test. It was obviously that the AgNW-RCF without HNO_3_ treatment exhibited an extremely low bonding strength because of the drastic increase of sheet resistance after 3 M tape test. The AgNW loss caused by 3 M tape test directly reduce the conductivity of the AgNW-RCF. In contrast, HNO_3_ treatment can improve the bonding strength between the AgNW and RCF. During the conductive film preparation, the AgNW was just precipitated onto the cellulose film; the AgNW was not tightly connected with the film substrate, leading to a relative low bonding strength. HNO_3_ treatment could make some melting effects that combine the AgNW and cellulose film together (Fig. 3c). From discuss mentioned above, HNO_3_ treatment not only can effectively enhance the optical transmittance, but also the bonding strength of the film.

The SEM and AFM images shown in Fig. 3a and d indicate that the RCF is almost smooth; the introduction of AgNW gives the combined film with a roughness surface (Fig. 3b, c, e and f). Wire junctions were apparently seen in the SEM image (Fig. 3b). After HNO_3_ (16%, 2 min) treatment, numbers of the wire junctions were removed (Fig. 3d and f). Fig. 3h and i shows the wire junction model before and after HNO_3_ treatment. Combined with the results from Fig. 2, it can be deduced that depletion of wire junctions could effectively enhance the conductivity and transmittance.

### 3.3 Properties of the perovskite solar cells fabricated on the AgNW-RCF

**Fig. 4:**
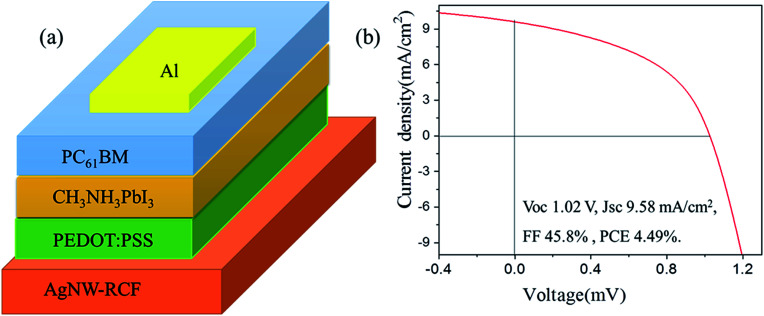
Structure diagram of perovskite thin film solar cell (a) and *J*–*V* curves of the devices (b).

In this part, the conductive cellulose film was used as an anode for perovskite solar cells fabrication, the structure diagram of the perovskite solar cell was shown in Fig. 4a. The cell shows a performance of *V*_oc_ 1.02 V, *J*_sc_ 9.58 mA cm^−2^, FF 45.8% and PCE 4.49% (Fig. 4b). Compared to the other reported data from perovskite solar cell, the solar cell exhibits extremely low photoelectric conversion efficiency (PCE).^28,29^ However, the efficiency of the solar cell was higher than that from solar cell (FTO–TiO_2_–perovskite-) based on AgNW/FTO and FTO^30^ and the CH_3_NH_3_PbI_3_-based photocell, which with spectral sensitivity only yielded a solar energy conversion efficiency of 3.8%.^9^ The probable reason for the low PCE of the solar cell based on the AgNW-RCF might be: (1) the fabrication process is not very suitable for AgNW-RCF. (2) The possible defects of the AgNW-RCF including relative low conductivity, surface roughness, shrink, thermal sensitivity might also account for the declined PCD. Anyway, the reasons are now under more detailed investigation. However, it is possible to use AgNW-RCF as an electrode in the perovskite solar cells in such a way that the fabrication process and the properties of AgNW-RCF are improved.

## 4 Conclusions

AgNWs synthesized by an ethylene glycol method were precipitated onto the RCF to impart the film with conductivity. The transmittance loss from AgNW introduction was diminished by HNO_3_ treatment; which can significantly enhance the optimal transmittance and bonding strength between AgNW and RCF without conductivity loss. As a result, photoelectric conversion efficiency was found to be 4.5% when the AgNW-RCF was incorporated in a perovskite solar cell.

## Conflicts of interest

There are no conflicts of interest to declare.

## Supplementary Material
